# Liver Fibrosis Estimated Using Noninvasive Blood Biochemical Indices Is Correlated with Visit-to-Visit Glycated Hemoglobin A1c Variability in Individuals with Type 2 Diabetes

**DOI:** 10.3390/biomedicines14051150

**Published:** 2026-05-19

**Authors:** Yousuke Kaneko, Taiki Hori, Kohsuke Miyataka, Takahito Asai, Tomoyo Hara, Hiroki Yamagami, Toshiki Otoda, Tomoyuki Yuasa, Akio Kuroda, Shingen Nakamura, Itsuro Endo, Munehide Matsuhisa, Ken-ichi Matsuoka, Ken-ichi Aihara

**Affiliations:** 1 Department of Internal Medicine, Tokushima Prefectural Kaifu Hospital, 266 Mugi-cho, Tokushima 775-0006, Japan; c202456028@tokushima-u.ac.jp; 2 Department of Hematology, Endocrinology and Metabolism, Graduate School of Biomedical Sciences, Tokushima University, 3-18-15 Kuramoto-cho, Tokushima 770-8503, Japan; hori.taiki@tokushima-u.ac.jp (T.H.); hara.tomoyo@tokushima-u.ac.jp (T.H.); yamagami.hiroki@tokushima-u.ac.jp (H.Y.); k-matsu@tokushima-u.ac.jp (K.-i.M.); 3 Department of Community Medicine and Medical Science, Graduate School of Biomedical Sciences, Tokushima University, 3-18-15 Kuramoto-cho, Tokushima 770-8503, Japan; asai.takahito@tokushima-u.ac.jp (T.A.); otoda.toshiki@tokushima-u.ac.jp (T.O.); yuasa.tomoyuki@tokushima-u.ac.jp (T.Y.); shingen@tokushima-u.ac.jp (S.N.); 4 Diabetes Therapeutics and Research Center, Institute of Advanced Medical Sciences, Tokushima University, 3-18-15 Kuramoto-cho, Tokushima 770-8503, Japan; kurodaakio@gmail.com (A.K.); matuhisa@tokushima-u.ac.jp (M.M.); 5 Department of Bioregulatory Sciences, Graduate School of Biomedical Sciences, Tokushima University, 3-18-15 Kuramoto-cho, Tokushima 770-8503, Japan; endoits@tokushima-u.ac.jp; 6 Department of Internal Medicine, Anan Medical Center, 6-1 Kawahara Takarada-cho, Anan 774-0045, Japan

**Keywords:** HbA1c variability, HbA1c-CV, MASLD, liver fibrosis

## Abstract

**Background/Objectives:** Visit-to-visit glycated hemoglobin A1c (HbA1c) variability is associated with cardiovascular diseases (CVDs) and all-cause mortality, independent of mean HbA1c levels. Metabolic dysfunction–associated steatotic liver disease (MASLD) is associated with CVDs and mortality. We aimed to clarify the association between annual HbA1c variability and MASLD development in individuals with type 2 diabetes (T2D). **Methods:** A retrospective cohort study was conducted in 402 Japanese patients (219 men, 183 women) with T2D. The participants’ HbA1c levels were measured every 2 months, and their HbA1c coefficient of variation (HbA1c-CV) was calculated from the HbA1c in the past year. We statistically evaluated the association between HbA1c-CV and noninvasive clinical indices of MASLD, including the hepatic steatosis index (HSI), fibrosis-4 (FIB-4) index, aspartate aminotransferase-to-platelet ratio index (APRI), and non-alcoholic fatty liver disease fibrosis score (NFS). **Results:** Multiple regression analysis of clinical variables and each MASLD index showed that all liver fibrosis indices, including the FIB-4 index (*p* < 0.001), APRI (*p* = 0.005), and NFS (*p* < 0.001), were positively correlated with HbA1c-CV, whereas the HSI was not (*p* = 0.148). These associations remained even after adjusting for the medications used in the participants. **Conclusions:** The development of liver fibrosis, estimated using noninvasive blood biochemical indices, is independently and positively associated with annual HbA1c-CV in individuals with T2D. This result suggests that a comprehensive approach, including early MASLD risk stratification, may be beneficial for optimal diabetes management.

## 1. Introduction

Diabetes mellitus (DM) is a risk factor for coronary heart disease and ischemic stroke, and the risks of mortality and diabetic complications increase among individuals with poor glycemic control [1]. Glycated hemoglobin A1c (HbA1c) is an indicator of blood glucose levels over 1–2 months and is commonly used in outpatient diabetic management. Management of diabetes, as assessed by HbA1c levels, is effective in reducing cardiovascular disease (CVD) mortality and the progression of diabetic microvascular complications [2,3]. Several guidelines, including those of the American Diabetes Association, have set target values for HbA1c, and diabetes management based on HbA1c is recommended, with some exceptions [4].

Glycemic variability is an important factor in the management of diabetes and its complications. Although the detailed mechanism is not yet clear, previous clinical studies have shown that visit-to-visit HbA1c variability is a risk factor for mortality, macrovascular complications, and diabetic microvascular complications, independent of HbA1c levels [5,6,7]. Previous studies have reported that the HbA1c-CV range was 2.0–3.0% in healthy individuals [8] and suggested that the risk of adverse outcomes increases when HbA1c-CV exceeds 5.0% [9].

In light of these circumstances, it has recently been considered important not only to maintain an average HbA1c level < 7% but also to minimize fluctuations in HbA1c levels between visits. However, the clinical factors affecting HbA1c fluctuations remain unknown.

Multiple factors, including biological stress, inflammation, overnutrition, and organ-derived humoral factors, form a complex network in the body that leads to the development of type 2 diabetes (T2D) and obesity. The liver, as the control center for glucose, protein, and lipid metabolism, plays an important role in regulating blood glucose fluctuations and maintaining homeostasis by dynamically changing gene expression in response to metabolic stress [10]. Metabolic dysfunction-associated steatotic liver disease (MASLD) is a crucial complication of diabetes that has attracted attention in recent years. MASLD is a newly defined steatotic liver disease with metabolic disorders, replacing non-alcoholic fatty liver disease (NAFLD) [11]. Approximately 40% of patients with MASLD progress to hepatic fibrosis [12], which leads to the development of liver cirrhosis and cancer [13]. In addition, a previous prospective cohort study showed that individuals with MASLD had higher overall and other cause-specific mortality risks than those without MASLD [14].

T2D is a risk factor for MASLD development, and 55.5% of individuals with T2D have MASLD [15]. Moreover, T2D and MASLD have adverse effects through the mechanisms of inflammation and insulin resistance [16].

Taken together, MASLD may influence blood glucose fluctuations, as indicated by HbA1c variability between visits; however, to date, studies on the association between MASLD severity and fluctuations in blood glucose control have not been conducted. Therefore, in this study, we aimed to examine the correlation between visit-to-visit HbA1c variability (annual HbA1c fluctuation) and MASLD development, as represented by noninvasive blood biochemical markers, in individuals with T2D.

## 2. Materials and Methods

### 2.1. Study Design and Data Source

We conducted a retrospective multicenter cohort study on outpatients from the Department of Internal Medicine at Tokushima Prefectural Kaifu Hospital and Anan Medical Center between January 2018 and March 2025. We obtained information on the clinical characteristics of 402 Japanese outpatients (219 men and 183 women) with T2D aged ≥ 20 years.

Information on the clinical characteristics and laboratory data of the participants was obtained using electronic medical records from each hospital, and all data were anonymized.

The participants were diagnosed with T2D based on a report by the Expert Committee on the Diagnosis and Classification of Diabetes Mellitus [17]. Participants who had smoked within the last 2 years were identified as current smokers. Participants with hypertension were defined as those with systolic blood pressure (BP) of ≥140 mmHg and/or diastolic BP of ≥90 mmHg or those who were taking antihypertensive drugs. Participants with dyslipidemia were defined as those who had a low-density lipoprotein cholesterol (LDL-C) level of ≥140 mg/dL, a fasting triglyceride (TG) level of ≥150 mg/dL or a casual TG level of ≥175 mg/dL, or a high-density lipoprotein cholesterol (HDL-C) level < 40 mg/dL or those who were receiving hypolipidemic drugs. Participants with exercise habits were identified as those who performed aerobic exercise, which is equivalent to walking for ≥30 min (≥3 metabolic equivalents of task) at least three times a week for at least 1 year.

This study excluded patients with steroid-induced or pancreatic diabetes, with advanced cancer, pregnant women, women consuming > 140 g of alcohol per week, men consuming > 210 g per week, and patients with liver cirrhosis or malnutrition (serum albumin [ALB] level < 3.0 g/dL). Patients with advanced renal disease and a blood creatinine (Cr) concentration of ≥ 2.0 mg/dL and those with a hemoglobin (Hgb) level < 10.0 g/dL or a hematocrit (Hct) level < 30% were also excluded. Finally, participants who had their medications added or changed during the 1-year observation period were also excluded. However, patients whose dosages of oral medications or insulin/GLP-1RA were changed were not excluded.

### 2.2. Glycated Hemoglobin A1c (HbA1c) Variability

We assessed HbA1c variability using the HbA1c coefficient of variation (HbA1c-CV) based on the HbA1c mean and standard deviation (SD), as outlined in a review by Monnier et al. [9]. HbA1c-CV is the ratio of the SD to the mean HbA1c level for each individual, following a common approach (Equation (1)):

CV = (HbA1c [SD])/HbA1c [mean]) × 100(1)

Generally, HbA1c levels are relatively high immediately after a diabetes diagnosis and fluctuate significantly with the initiation of treatment, including insulin use, after diagnosis. To eliminate these initial fluctuations of HbA1c levels, this study included only participants who had been receiving treatment for T2D for more than three years. Among these individuals with T2D, we targeted participants who regularly visited our hospitals and had their HbA1c levels measured every 2 months. The mean and SD of HbA1c levels were calculated from seven HbA1c values measured over 1 year from the date of the last visit.

### 2.3. Biochemical Analysis

Blood cell counts; plasma glucose (PG); HbA1c; and serum biochemical parameters, including LDL-C, TG, HDL-C, uric acid (UA), aspartate aminotransferase (AST), alanine aminotransferase (ALT), Cr, and ALB, were included in the analyses. PG and serum levels of LDL-C, TG, HDL-C, UA, AST, ALT, Cr, and ALB were measured using enzymatic methods. HbA1c levels were assayed using high-performance liquid chromatography.

### 2.4. Determination of Severity of Steatotic Liver Disease Represented by Noninvasive Blood Chemical Biomarkers

The hepatic steatosis index (HSI), a specific marker of fatty liver, was calculated for each participant using the following formula: 8 × (ALT/AST ratio) + body mass index (BMI) (+2 if female, +2 if diabetes was present). The presence of fatty liver was suggested when it was >36 [18].

We also used three clinical scoring systems, the fibrosis-4 (FIB-4) index, AST-to-platelet ratio index (APRI), and NAFLD fibrosis score (NFS), to estimate liver fibrosis. Each index was determined using the following formula:

∗ FIB-4 index = age (years) × AST (U/L)/(platelet count [×10^9^/L] × √ALT [U/L]) [19]
∗ APRI = 100 × AST (U/L)/upper limit of normal AST (U/L)/platelet count (×10^9^/L) [20]
∗ NFS = −1.675 + 0.037 × age (years) + 0.094 × BMI (kg/m^2^) + 1.13 × IFG/diabetes (yes = 1, no = 0) + 0.99 × AST/ALT ratio − 0.013 × platelet count (×10^9^/L) − 0.66 × ALB (g/dL) [21].

While liver biopsy remains a standard diagnostic method for evaluating the staging of liver fibrosis in patients with MASLD, it is far too invasive to be the first-line screening method for a large number of patients. Furthermore, histological lesions are unevenly distributed throughout the liver tissue, leading to a significant problem of variability in pathological diagnosis depending on the sampling site. As a result, it has been reported that relying entirely on liver biopsy carries the risk of underestimating the severity of liver fibrosis [22]. On the other hand, noninvasive scoring systems, including the FIB-4 index, APRI, and NFS, have demonstrated the ability to categorize the severity of hepatic fibrosis comparably to or even superiorly to that of liver biopsy [23,24].

### 2.5. Statistical Analysis

Using the Shapiro–Wilk test, the normality of the distribution of continuous variables was determined. Means and standard deviation (SD) were used to express continuous variables with a normal distribution, while medians (Q1, Q3) were used to express those with a non-normal distribution. Numbers or percentages were used to express categorical parameters. Dummy variables were assigned to males; subjects who had diabetes, hypertension, or dyslipidemia; and subjects who were current smokers. Multivariate regression and logistic regression analyses were used to determine the relationships between clinical markers of fatty liver (HSI) or liver fibrosis (FIB-4 index, APRI, and NFS) and clinical variables, including HbA1c-CV. All of the analyses were conducted using GraphPad Prism 10 (version 10.6.1) (GraphPad Software, San Diego, CA, USA). The threshold for statistical significance was set as *p* < 0.05.

## 3. Results

### 3.1. Characteristics of the Participants

The physical and laboratory characteristics of the participants included in this study are shown in Table 1. The male participants showed higher casual PG levels and higher serum levels of UA, Cr, AST, and ALT than the female participants. The median HbA1c level was 6.9%, and the median HbA1c-CV was 3.8%, with no sex differences observed in either parameter. No significant sex differences were observed in the HSI, but the male participants showed significantly higher values in liver fibrosis indices, including the FIB-4 index (*p* = 0.015), APRI (*p* < 0.001), and NFS (*p* = 0.005), than the female participants. The rates of current smoking and exercise habits were significantly higher in the male participants than in the female participants. The percentage of female participants using statins was higher than that of the male participants. The percentage of the male participants using antiplatelet therapy was higher than that of the female participants.

### 3.2. Association Between HbA1c Coefficient of Variation and Clinical Markers of MASLD

**Figure 1:**
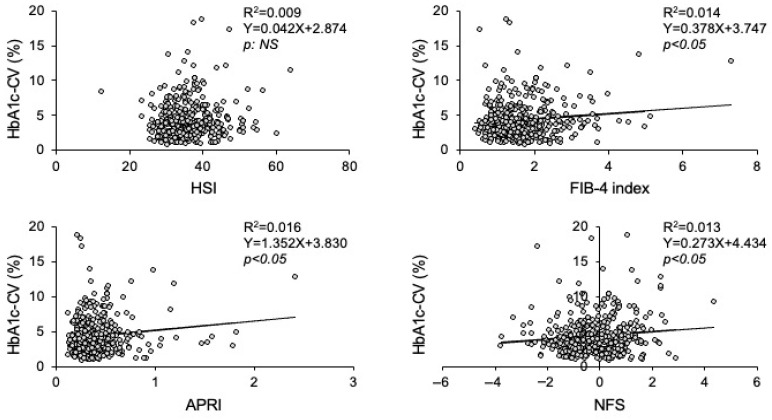
Scatter plots between HbA1c-CV and MASLD-associated indices.

In simple regression analysis, HSI had no statistical correlation with HbA1c-CV (*p* = 0.053) (Figure 1). On the other hand, the FIB-4 index (*p* = 0.019), APRI (*p* = 0.012) and NFS (*p* = 0.023) showed significant positive correlations with HbA1c-CV (Figure 1).

Although multiple regression analysis showed no significant correlation between the HSI and HbA1c-CV (*p* = 0.148) (Table 2 and Table S1), all liver fibrosis indices, including the FIB-4 index (*p* < 0.001), APRI (*p* = 0.002), and NFS (*p* < 0.001), were positively correlated with HbA1c-CV (Table 2). In addition, baseline HbA1c level and current smoking status were independently and positively correlated with HbA1c-CV. In the multiple regression analysis to determine HbA1c-CV-associated medications, insulin (*p* < 0.001) and glucagon-like peptide-1 receptor agonists (*p* = 0.004) were found to be positively correlated (Table 3).

Finally, we conducted multiple regression analyses using correlated clinical factors and medications and verified that all three liver fibrosis indices had independent and positive correlations with HbA1c-CV. (FIB-4 index, *p* < 0.001; APRI, *p* = 0.005; NFS, *p* < 0.001) (Table 4).

## 4. Discussion

In this study, we found that baseline liver fibrosis indices were positively correlated with annual HbA1c fluctuation, represented by HbA1c-CV, suggesting that liver fibrosis is associated with the development of diabetes-related complications and poor prognosis.

HbA1c levels fluctuate depending on various factors, including insufficient exercise, dietary habits, and medication compliance. Specifically, the factors reported to increase HbA1c-CV in individuals with T2D include skipping breakfast [25], male sex, younger age, and higher BMI [26,27]. In addition, previous studies have reported that participants with a high HbA1c-CV tend to have high HbA1c values, similar to our results [27,28].

To ensure proper management of HbA1c levels and avoid extreme hyperglycemia and hypoglycemia, it is crucial to thoroughly analyze the factors that affect HbA1c-CV. Therefore, it is necessary to determine why liver fibrosis, a risk factor discovered in this study, is associated with increased HbA1c variability.

There are several possible explanations for the mechanism by which liver fibrosis contributes to an increase in HbA1c-CV.

First, HbA1c levels are affected by fluctuations in red blood cell (RBC) counts and lifespan. In individuals with diabetes and iron deficiency anemia (IDA), HbA1c levels are significantly lower than those in individuals with diabetes without IDA [29]. In advanced chronic liver disease (CLD) such as liver cirrhosis, abnormalities in the RBC membrane due to lipid metabolism disorders and oxidative stress and hypersplenism shorten the lifespan of RBCs [30,31,32]. Therefore, the shortening of the RBC lifespan due to the progression of liver fibrosis may be linked to increased HbA1c-CV levels.

Second, patients with liver fibrosis are prone to blood glucose fluctuations owing to the following mechanisms. Patients with CLD experience nocturnal hypoglycemia due to decreased hepatic glycogen storage and impaired gluconeogenesis [33]. However, these patients also experience postprandial hyperglycemia through various mechanisms, such as the formation of portosystemic shunts and increased insulin resistance [34]. Additionally, oxidative stress creates a vicious cycle between liver fibrosis and glycemic control. Oxidative stress plays a central role in the progression of fatty liver disease to metabolic dysfunction-associated steatohepatitis, causing lipid peroxidation, inflammation, and fibrosis through an imbalance between reactive oxygen species (ROS) and antioxidant defenses [35]. Specifically, the progression of liver fibrosis is positively correlated with increased oxidative stress, and this correlation is expressed using the pro-oxidant–antioxidant balance method [36]. Furthermore, oxidative stress contributes to blood glucose fluctuations via increased insulin resistance and reduced insulin secretion due to pancreatic β-cell damage [37,38]. HbA1c-CV has been shown to be inversely correlated with the time in range (TIR) of the daily glucose profile in individuals with T2D [39], and it has been reported that the FIB-4 index and NFS exhibit an inverse correlation with TIR in participants with T2D [40]. These findings suggest that the progression of liver fibrosis is associated with an increase in daily blood glucose variability, which could be associated with long-term HbA1c-CV.

On the other hand, HbA1c-CV has been reported as an independent risk factor for the development of MASLD in individuals with T2D [41]. Furthermore, HbA1c-CV has been shown to be positively correlated with subclinical inflammatory markers such as C-reactive protein and ferritin and to exacerbate insulin resistance estimated by HOMA-IR [41]. In addition, short-term blood glucose fluctuations, such as daily variation, have been reported to increase oxidative stress and inflammation in hepatocytes [42,43]. Therefore, it is suggested that the increase in HbA1c-CV, an indicator based on fluctuations between medical visits, and the progression of MASLD are closely related through a bidirectional and common pathological mechanism.

**Figure 2:**
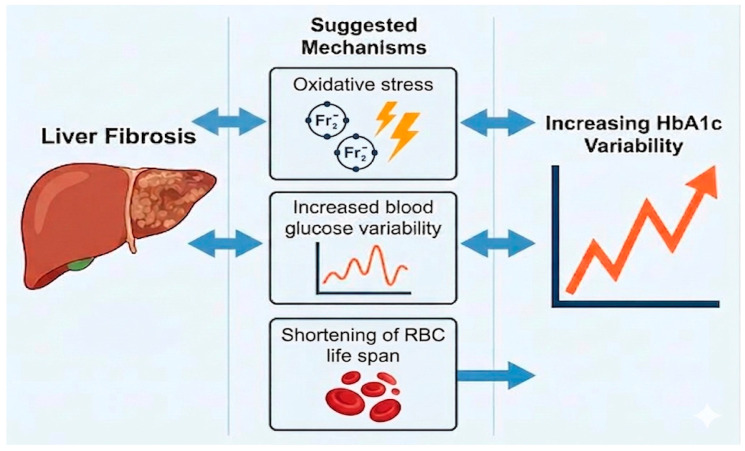
Summary illustration of the association between the increased HbA1c variability and liver fibrosis with suggested mechanisms in individuals with T2D.

Figure 2 illustrates the suggested mechanisms that link liver fibrosis to increased HbA1c variability.

In this study, current smoking was a significant risk factor for increased HbA1c-CV in individuals with T2D. Active smoking was associated with an increased risk of incident T2D, and quitting smoking decreased the risk of diabetes in the long term [44]. Smoking increases inflammation and oxidative stress, leading to the development of insulin resistance [45]. Nicotine increases ROS production during metabolism and inhibits nuclear factor erythroid 2-related factor 2, increasing oxidative stress and inducing insulin resistance [46]. Moreover, nicotine activates mammalian target of rapamycin and phosphorylates insulin receptor substrate 1, thereby reducing insulin sensitivity and increasing insulin resistance [47].

In the present study, multivariate analysis between medications used and HbA1c-CV showed that both insulin and GLP-1RAs were independently positively correlated with HbA1c-CV; however, this association disappeared after adjusting for significant clinical factors. Several cross-sectional studies have suggested a positive correlation between insulin use and liver fibrosis in NAFLD patients [48,49]. Contrastingly, GLP-1RAs have recently been reported to improve liver fibrosis [50]; however, our study showed a positive correlation between GLP-1RA use and HbA1c fluctuations. These results suggest being a result of GLP-1RAs being prescribed more often to patients with severe metabolic dysfunction or advanced MASLD. The initial correlation between these drugs and HbA1c-CV may reflect confounding due to disease severity rather than the direct effect of the drugs themselves. Consequently, we believe that insulin and/or GLP-1RAs are necessary in many diabetic individuals with liver fibrosis to optimize blood glucose management.

In addition, severity of fatty liver, represented by HSI, did not show a significant correlation with HbA1c-CV in this study. Our previous study has shown significant correlation of liver fibrosis indices (FIB-4 index, APRI and NFS) but not HSI with MCV of red blood cells, a marker of age-related changes [51]. These findings suggest that fatty liver has a fundamentally different pathological condition from liver fibrosis in terms of aging and metabolic disorders.

### Limitations

This study has some limitations. First, liver fibrosis was not evaluated using liver biopsy or imaging examinations, such as ultrasonography or magnetic resonance imaging. The reliability of our hypothesis could be enhanced by evaluating the association between HbA1c-CV and liver fibrosis using liver biopsy or noninvasive imaging examinations. Second, this study was based on a retrospective cohort, and there were unmeasured confounding factors, such as inflammation and oxidative stress biomarkers, which could not be evaluated. Therefore, it is imperative to interpret the causal association between liver fibrosis and HbA1c-CV with caution. Third, this study may be subject to selection bias because it included only clinically stable patients. Furthermore, it should be noted that the impact of this bias may be complicated by the fact that participants who required changes or additions to their medication during the observation period were excluded, while participants whose medication remained unchanged but whose dosage was altered were not excluded.

## 5. Conclusions and Perspectives

The development of liver fibrosis, estimated using noninvasive blood biochemical indices, is independently and positively associated with annual HbA1c-CV in individuals with T2D. These results suggest that a comprehensive approach, including early MASLD risk stratification, may be beneficial for optimal diabetes management. Conversely, in patients with greater HbA1c fluctuations, the possibility of liver fibrosis should be carefully considered.

Since our findings in this study, along with previously published research, suggest a bidirectional relationship between liver fibrosis and fluctuations in blood glucose management, the causal relationship between the two factors remains unclear. Therefore, large-scale prospective intervention studies are needed to determine whether preventing liver fibrosis leads to a reduction in CVDs and mortality through stabilization of glycemic control.

## Figures and Tables

**Table 1:** Clinical characteristics of the participants.

Clinical Factors	Total	Male	Female	*p*-Value
Number of participants	402	219	183	
Age (years)	72 (63–77)	72 (62–77)	71 (64–76)	0.678
BMI (kg/m^2^)	24.3 (21.9–26.9)	24.6 (22.3–26.6)	24.2 (21.7–27.2)	0.430
SBP (mmHg)	134.6 ± 17.7	134.0 ± 14.5	135.3 ± 20.8	0.462
LDL-C (mg/dL)	98.0 (80.0–118.0)	97.0 (79.0–118.5)	99.0 (81.2–118.0)	0.653
TG (mg/dL)	112.5 (86–165.8)	117.0 (87.0–170.0)	110.0 (83.0–156.5)	0.215
HDL-C (mg/dL)	53.9 (44.0–63.0)	51.0 (41.2–60.2)	57.8 (49.0–66.7)	<0.001
Casual PG (mg/dL)	137.0 (117.0–173.0)	147.0 (124.0–192.5)	128.0 (113.0–157.5)	0.002
HbA1c (%)	6.9 (6.5–7.4)	6.9 (6.5–7.4)	6.9 (6.6–7.4)	0.615
HbA1c-CV (%)	3.8 (2.6–5.2)	4.0 (2.6–5.5)	3.6 (2.5–4.9)	0.964
UA (mg/dL)	5.1 (4.2–5.9)	5.4 (4.6–6.2)	4.5 (3.8–5.5)	<0.001
Cr (mg/dL)	0.77 (0.63–0.96)	0.88 (0.75–1.06)	0.64 (0.56–0.77)	<0.001
eGFR (mL/min/1.73 m^2^)	68.7 ± 19.4	67.5 ± 20.0	70.1 ± 18.5	0.180
ALB (g/dL)	4.2 ± 0.3	4.2 ± 0.3	4.2 ± 0.3	0.355
AST (U/L)	21 (17.0–26.0)	21 (17.0–28.0)	20 (17.0–25.0)	0.039
ALT (U/L)	21.0 (15.0–29.8)	21.0 (16.0–32.0)	19.0 (13.0–26.5)	0.001
RBC (10^12^/L)	4.62 (4.28–5.00)	4.77 (4.36–5.15)	4.55 (4.21–4.82)	0.035
Hct (%)	42.3 (39.4–45.1)	43.5 (40.9–46.3)	41.2 (38.3–43.2)	<0.001
Hgb (g/dL)	14.0 (12.9–15.1)	14.5 (13.7–15.7)	13.5 (12.5–14.3)	<0.001
MCV (fL)	91.4 (88.8–94.7)	91.9 (88.9–95.2)	90.7 (88.7–93.6)	0.063
Platelets (10^9^/L)	216.0 (181.0–254.0)	209.0 (173.0–235.0)	232.0 (198.0–267.5)	<0.001
HSI	34.9 (31.9–39.0)	34.5 (32.0–38.2)	36.0 (31.9–39.9)	0.259
FIB-4 index	1.52 (1.10–2.05)	1.61 (1.13–2.17)	1.42 (1.08–1.92)	0.015
APRI	0.35 (0.26–0.48)	0.39 (0.28–0.53)	0.32 (0.24–0.40)	<0.001
NFS	−0.15 (−0.95–0.53)	0.05 (−0.82–0.67)	−0.40 (−1.08–0.40)	0.005
Exercise habit (*n*, [%])	105 (26.1)	70 (32.0)	35 (19.1)	0.004
Current smoker (*n*, [%])	50 (12.4)	48 (21.9)	2 (1.1)	<0.001
Hypertension (*n*, [%])	287 (71.4)	151 (68.9)	136 (74.3)	0.236
Dyslipidemia (*n*, [%])	301 (74.9)	161 (73.5)	140 (76.5)	0.492
Duration of T2D (years)	11.0 (6.0–19.0)	11.0 (6.0–17.0)	11.0 (7.5–20.0)	0.159
ARB or ACEi (*n*, [%])	215 (53.5)	113 (51.6)	102 (55.7)	0.407
CCB (*n*, [%])	193 (48.0)	101 (46.1)	92 (50.3)	0.406
β blocker (*n*, [%])	30 (7.5)	24 (11.1)	6 (3.3)	0.004
MR antagonist (*n*, [%])	5 (1.2)	3 (1.4)	2 (1.1)	0.803
Statin (*n*, [%])	219 (54.5)	104 (47.5)	115 (62.8)	0.002
Ezetimibe (*n*, [%])	45 (11.2)	26 (11.9)	19 (10.4)	0.637
Antiplatelet (*n*, [%])	58 (14.4)	44 (20.1)	14 (7.7)	<0.001
SU or glinide (*n*, [%])	103 (25.6)	52 (23.7)	51 (27.9)	0.345
Metformin (*n*, [%])	212 (52.7)	114 (52.1)	98 (53.6)	0.765
DPP-4i (*n*, [%])	266 (66.2)	139 (63.5)	127 (69.4)	0.211
SGLT2i (*n*, [%])	161 (40.0)	92 (42.0)	69 (37.7)	0.380
α-GI (*n*, [%])	77 (19.2)	48 (21.9)	29 (15.8)	0.123
Pioglitazone (*n*, [%])	16 (4.0)	8 (3.7)	8 (4.4)	0.714
Insulin (*n*, [%)])	81 (20.1)	45 (20.5)	36 (19.7)	0.827
GLP-1RA (*n*, [%)])	44 (10.9)	25 (11.4)	19 (10.4)	0.741

Values are presented as means ± SDs or medians (Q1, Q3). Abbreviations: BMI, body mass index; SBP, systolic blood pressure; LDL-C, low-density lipoprotein cholesterol; TG, triglyceride; HDL-C, high-density lipoprotein cholesterol; PG, plasma glucose; HbA1c, hemoglobin A1c; CV, coefficient of variation; UA, uric acid; Cr, creatinine; eGFR, estimated glomerular filtration rate; ALB, albumin; AST, aspartate aminotransferase; ALT, alanine aminotransferase; RBC, red blood cell; Hct, hematocrit; Hgb, hemoglobin; MCV, mean corpuscular volume; HSI, hepatic steatosis index; FIB-4, fibrosis-4 index; APRI, aspartate aminotransferase-to-platelet ratio index; NFS, NAFLD fibrosis score; ARB, angiotensin II receptor blocker; ACEi, angiotensin-converting enzyme inhibitor; CCB, calcium channel blocker; MR, mineralocorticoid receptor; SU, sulfonylurea; DPP-4i, dipeptidyl peptidase-4 inhibitor; SGLT2i, sodium-glucose cotransporter-2 inhibitor; α-GI, alpha-glucosidase inhibitor; GLP-1RA, glucagon-like peptide-1 receptor agonist.

**Table 2:** Multiple regression analysis of clinical factors for the determinants of HbA1c-CV.

	HbA1c-CV											
	HSI			FIB-4 Index			APRI			NFS		
Variables	VIF	*t*-Value	*p*-Value	VIF	*t*-Value	*p*-Value	VIF	*t*-Value	*p*-Value	VIF	*t*-Value	*p*-Value
Age	1.855	−0.85	0.397	1.947	−1.94	0.053	1.711	−0.58	0.565	2.478	−2.38	0.018
Male	1.734	−1.13	0.260	1.743	−1.57	0.117	1.769	−1.53	0.128	1.750	−1.51	0.132
BMI	6.895	2.11	0.036	1.658	2.04	0.042	1.651	1.68	0.093	1.846	0.49	0.622
SBP	1.082	−1.27	0.207	1.155	−1.23	0.218	1.156	−1.21	0.229	1.156	−1.35	0.179
LDL-C	1.273	1.17	0.244	1.115	1.50	0.136	1.109	1.27	0.204	1.135	1.65	0.101
TG	1.156	1.10	0.272	1.365	1.11	0.269	1.364	1.01	0.313	1.381	1.34	0.181
HDL-C	1.390	0.39	0.694	1.392	0.56	0.556	1.393	0.55	0.583	1.395	0.64	0.526
HbA1c	1.111	9.41	<0.001	1.181	9.83	<0.001	1.178	9.59	<0.001	1.181	9.71	<0.001
UA	1.424	−0.07	0.947	1.420	0.17	0.862	1.419	−0.05	0.964	1.434	0.40	0.687
Cr	1.753	1.28	0.201	1.746	1.65	0.099	1.767	1.79	0.075	1.746	1.23	0.218
ALB	1.206	−2.25	0.025	1.201	−2.00	0.046	1.190	−2.27	0.024	1.331	−1.18	0.240
Hct	1.493	−0.23	0.816	1.484	−0.47	0.638	1.487	−0.51	0.613	1.484	−0.44	0.660
Exercise habit	1.082	1.18	0.239	1.081	1.17	0.244	1.081	1.34	0.182	1.082	1.12	0.264
Current Smoking	1.273	2.08	0.038	1.263	2.40	0.017	1.262	2.33	0.020	1.272	2.57	0.011
Hypertension	1.267	1.04	0.297	1.269	1.25	0.211	1.268	1.15	0.253	1.290	1.53	0.127
Dyslipidemia	1.183	−0.84	0.404	1.181	−0.92	0.360	1.179	−0.74	0.462	1.183	−0.96	0.338
Duration of T2D	1.259	−0.36	0.717	1.255	−0.39	0.700	1.256	−0.35	0.726	1.256	−0.58	0.565
HSI	7.657	−1.45	0.148	-	-	-	-	-	-	-	-	-
FIB-4 index	-	-	-	1.369	4.29	<0.001	-	-	-	-	-	-
APRI	-	-	-	-	-	-	1.073	3.12	0.002	-	-	-
NFS	-	-	-	-	-	-	-	-	-	2.112	3.57	<0.001

VIF, variance inflation factor.

**Table 3:** Multiple regression analysis of medications for the determinants of HbA1c-CV.

	HbA1c-CV		
Variables	VIF	*t*-Value	*p*-Value
ARB or ACEi	1.282	0.71	0.479
CCB	1.292	−0.10	0.919
β blocker	1.189	0.54	0.588
MR antagonist	1.107	0.18	0.858
Statin	1.088	−1.03	0.303
Ezetimibe	1.212	−1.00	0.316
Antiplatelet	1.131	−0.16	0.872
SU or glinide	1.119	1.62	0.107
Metformin	1.103	1.78	0.077
DPP-4i	1.479	1.50	0.134
SGLT2i	1.079	0.06	0.956
α-GI	1.109	−0.37	0.709
Pioglitazone	1.049	1.95	0.052
Insulin	1.105	3.56	<0.001
GLP-1RA	1.456	2.91	0.004

**Table 4:** Total multiple regression analysis of correlated clinical factors and medications for the determinants of HbA1c-CV.

	HbA1c-CV								
	FIB-4 Index			APRI			NFS		
Variables	VIF	*t*-Value	*p*-Value	VIF	*t*-Value	*p*-Value	VIF	*t*-Value	*p*-Value
Age	-	-	-	-	-	-	1.487	−2.61	0.010
BMI	1.104	3.17	0.002	-	-	-	-	-	-
HbA1c	1.136	9.46	<0.001	1.122	9.43	<0.001	1.139	9.51	<0.001
ALB	1.075	−1.86	0.064	1.027	−2.20	0.029	-	-	-
Current smoking	1.043	2.08	0.038	1.033	1.54	0.126	1.046	1.63	0.105
Insulin	1.148	0.82	0.413	1.142	0.64	0.521	1.125	0.97	0.332
GLP-1RA	1.071	1.57	0.118	1.061	1.56	0.120	1.074	1.52	0.131
FIB-4 index	1.129	3.52	<0.001	-	-	-	-	-	-
APRI	-	-	-	1.011	2.82	0.005	-	-	-
NFS	-	-	-	-	-	-	1.488	3.94	<0.001

## Data Availability

Data supporting the findings of this study are available upon request from the corresponding author. The data are not accessible to the public because of privacy and ethical restrictions.

## Supplementary Materials

The following supporting information can be downloaded at: https://www.mdpi.com/article/10.3390/biomedicines14051150/s1. Table S1. Multiple regression analysis of clinical factors (including HSI, without BMI) for the determinants of HbA1c-CV.

## Abbreviations

HbA1c	glycated hemoglobin A1c
CVDs	cardiovascular diseases
MASLD	metabolic dysfunction–associated steatotic liver disease
T2D	type 2 diabetes
HbA1c-CV	HbA1c coefficient of variation
HSI	hepatic steatosis index
FIB-4	fibrosis-4
APRI	aspartate aminotransferase-to-platelet ratio index
NAFLD	non-alcoholic fatty liver disease
NFS	non-alcoholic fatty liver disease fibrosis score
BP	blood pressure
LDL-C	low-density lipoprotein cholesterol
TG	triglyceride
HDL-C	high-density lipoprotein cholesterol
ALB	albumin
Cr	creatinine
Hgb	hemoglobin
SD	standard deviation
PG	plasma glucose
UA	uric acid
AST	aspartate aminotransferase
BMI	body mass index
RBC	red blood cell
IDA	iron deficiency anemia
CLD	chronic liver disease
ROS	reactive oxygen species
TIR	time in range
