# Supplementary material for: Liver Fibrosis Estimated Using Noninvasive Blood Biochemical Indices Is Correlated with Visit-to-Visit Glycated Hemoglobin A1c Variability in Individuals with Type 2 Diabetes
Source: Biomedicines. 2026 May 19;14(5):1150. doi: 10.3390/biomedicines14051150 (PMC13204113; doi:10.3390/biomedicines14051150)
Supplement: Supplementary file 1 [file biomedicines-14-01150-s001.zip › biomedicines-4193064-supplementary.pdf]

Table S1. Multiple regression analysis of clinical factors (including HSI, without BMI) for the determinants of HbA1c-CV.

		**HbA1c-CV**	
Variables	VIF	*t* value	*p* value
Age	1.840	–0.66	0.353
Male	1.724	–0.97	0.334
SBP	1.151	–1.13	0.261
LDL-C	1.097	0.93	0.352
TG	1.366	0.87	0.387
HDL-C	1.372	0.15	0.878
HbA1c	1.177	9.30	<0.001
UA	1.399	0.21	0.831
Cr	1.742	1.45	0.148
ALB	1.180	–2.58	0.010
Hct	1.490	–0.14	0.893
Exercise habit	1.082	1.15	0.249
Current Smoking	1.273	2.04	0.042
Hypertension	1.261	1.18	0.240
Dyslipidemia	1.182	–0.77	0.444
Duration of T2D	1.235	–0.66	0.509
HSI	1.833	0.79	0.429
